# Development of marker-free transgenic *Jatropha curcas* producing curcin-deficient seeds through endosperm-specific RNAi-mediated gene silencing

**DOI:** 10.1186/s12870-015-0625-z

**Published:** 2015-10-08

**Authors:** Keyu Gu, Dongsheng Tian, Huizhu Mao, Lifang Wu, Zhongchao Yin

**Affiliations:** Temasek Life Sciences Laboratory, 1 Research Link, National University of Singapore, Singapore, 117604 Republic of Singapore; Department of Biological Sciences, National University of Singapore, 14 Science Drive, Singapore, 117543 Republic of Singapore; School of Biological Sciences, Nanyang Technological University, 60 Nanyang Drive, Singapore, 637551 Republic of Singapore; Present address: Hefei Institutes of Physical Science, Chinese Academy of Sciences, Hefei, 230031 Anhui China

**Keywords:** *Jatropha curcas*, Curcin, RNAi, Marker-free transformation, Gene silencing, Detoxification

## Abstract

**Background:**

*Jatropha curcas* L. is a potential biofuel plant and its seed oil is suitable for biodiesel production. Despite this promising application, jatropha seeds contain two major toxic components, namely phorbol esters and curcins. These compounds would reduce commercial value of seed cake and raise safety and environment concerns on jatropha plantation and processing. Curcins are Type I ribosome inactivating proteins. Several curcin genes have been identified in the jatropha genome. Among which, the *Curcin 1* (*C1*) gene is identified to be specifically expressed in endosperm, whereas the *Curcin 2A* (*C2A*) is mainly expressed in young leaves.

**Results:**

A marker-free RNAi construct carrying a *β*-estradiol-regulated Cre/*loxP* system and a *C1* promoter-driven RNAi cassette for *C1* gene was made and used to generate marker-free transgenic RNAi plants to specifically silence the *C1* gene in the endosperm of *J. curcas*. Plants of transgenic line L1, derived from T0-1, carry two copies of marker-free RNAi cassette, whereas plants of L35, derived from T0-35, harbored one copy of marker-free RNAi cassette and three copies of closely linked and yet truncated *Hpt* genes. The C1 protein content in endosperm of L1 and L35 seeds was greatly reduced or undetectable, while the C2A proteins in young leaves of T0-1 and T0-35 plants were unaffected. In addition, the *C1* mRNA transcripts were undetectable in the endosperm of T3 seeds of L1 and L35. The results demonstrated that the expression of the *C1* gene was specifically down-regulated or silenced by the double-stranded RNA-mediated RNA interference generated from the RNAi cassette.

**Conclusion:**

The *C1* promoter-driven RNAi cassette for the *C1* gene in transgenic plants was functional and heritable. Both *C1* transcripts and C1 proteins were greatly down-regulated or silenced in the endosperm of transgenic *J. curcas*. The marker-free transgenic plants and curcin-deficient seeds developed in this study provided a solution for the toxicity of curcins in jatropha seeds and addressed the safety concerns of the marker genes in transgenic plants on the environments.

## Background

Jatropha (*Jatropha curcas* L.) is a potential oilseed crop for the production of renewable bioenergy [1]. However, jatropha seeds contain toxic and anti-nutritive compounds, which include phorbol esters, curcins, saponins, trypsin inhibitors, protease inhibitors, curcain, jatrophidin, phytates, alkaloids, lectins, lignans, tannins, latex and cyclic peptides [2]. The presence of these compounds in jatropha seeds renders the seedcake for being unsuitable for animal feed and raises safety and environment concerns on jatropha plantation and processing [3, 4].

Ribosome-inactivating proteins (RIPs) are found in many plants, fungi and bacteria. They are toxic *N*-glycosidases that depurinate the universally conserved α-sarcin loop of large rRNAs, which inactivates the ribosome, thereby blocking its further participation in protein synthesis [5, 6]. Curcins in *J. curcas* belong to Type I RIPs, which are common among the members of the Euphobiaceae family. Curcin is analogous to ricin, a Type II RIP, in *Ricinus communis*. However, the toxicity of curcin is significantly lower than that of ricin [7, 8]. The biochemical function of curcin in *J. curcas* is not well known and several reports suggest that it may play a role in defense against biotic and abiotic stress [9–12]. Besides, curcins were also found to show antitumor activity and have promising potential in cancer therapy [13–17].

More than 10 curcin genes have been isolated from different jatropha accessions and the amino acid sequences of the deduced curcin proteins are available in Genbank. Members of curcins share at least 86 % identity at amino acid level. These curcin proteins can be classified into two types. Type-I curcins have a precursor of 293 amino acid residues and a mature protein of about 28 kilo-dalton (kDa) and were only identified in jatropha seeds [4, 8, 18, 19]. Type-II curcins have a precursor of 309 amino acid residues and a mature protein of about 30 kDa [10, 12]. They were mainly found to be present in jatropha leaves and some of which were induced by abiotic stress [10, 12]. The whole genome sequencing of *J. curcas* indicates that there are three curcin genes and two additional curcin-like genes in the jatropha genome [20]. In a companion article, we report the isolation of one Type-I curcin gene, *Curcin 1* (*C1*), and two Type-II curcin genes, *Curcin 2A* (*C2A*) and *Curcin 2B* (*C2B*), from *J. curcas* MD44, an elite Indonesia accession. *C1* and *C2A* are expressed in developing seeds and young leaves, respectively. However, no *C2B* transcripts were detected in developing seeds and leaves of *J. curcas*.

Selectable marker genes usually confer antibiotic or herbicide resistance for the selection of transformants during plant transformation. Their removal, would eliminate potential environmental and health-related risks and technical barriers for the subsequent rounds of plant transformation. In addition, production of marker-free transgenic plants would increase the consumer acceptance of genetically modified crops and their products. Zuo et al. (2001) developed a chemically regulated and Cre/*loxP*-mediated recombination system for marker-free transformation in Arabidopsis. In this system, the expression of the *Cre* gene is controlled by an estrogen receptor-based fusion transactivator XVE, which is activated by the addition of *β*-estradiol [21]. We successfully adopted this chemically regulated, Cre/*loxP*-mediated marker-free transformation system in rice [22, 23] and *J. curcas* [24].

Here we report the development of marker-free transgenic jatropha plants and *C1* promoter-driven endosperm-specific RNAi mediated *C1* gene silencing in jatropha seeds. Curcin-free jatropha seeds help to detoxify the seedcake as animal feed and address safety concerns on jatropha plantation and seed processing.

## Results

### Generation of transgenic jatropha plants that produced T1 seeds with low curcin content

**Fig. 1:**
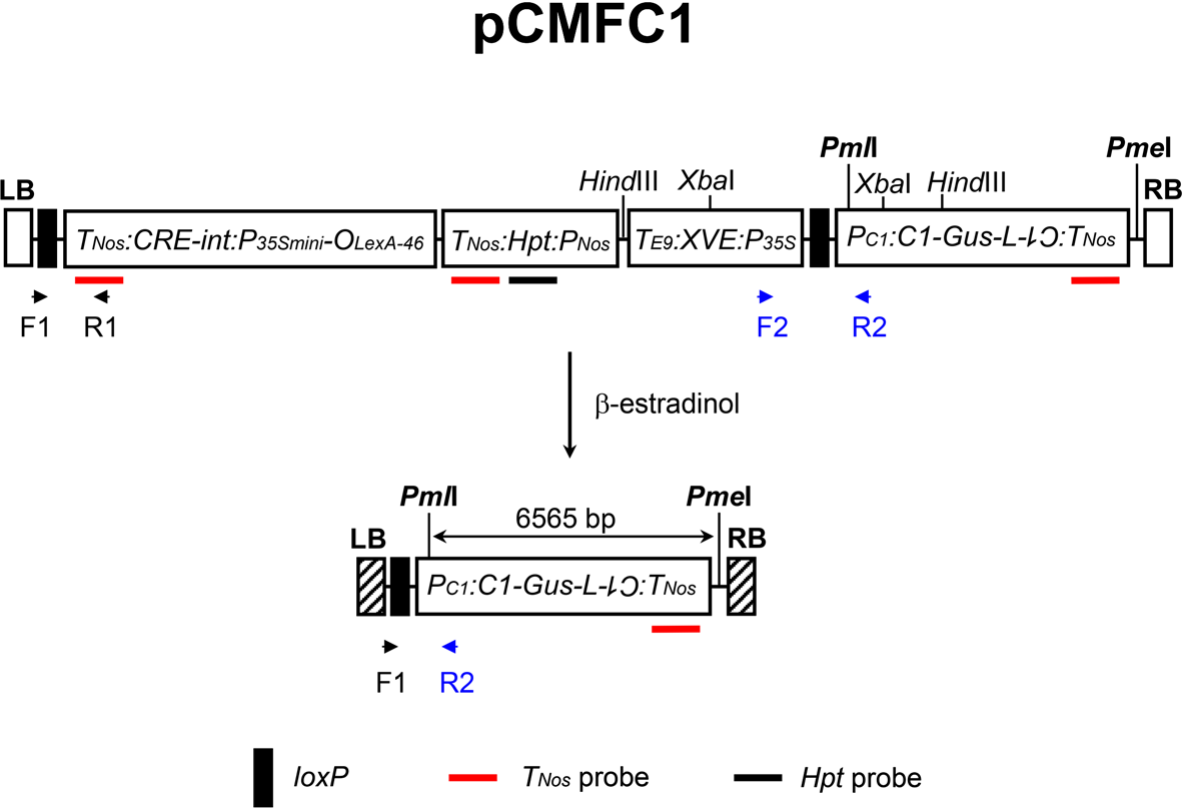
A schematic diagram of the T-DNA region of the construct pCMFC1 and Cre/*loxP*-mediated DNA recombination (*Map not drawn to scale*). Region flanked by the two *loxP* sites (filled boxes) in the upper diagram is the *loxP* fragment, which is excised by Cre/*loxP*-mediated DNA recombination after *β*-estradiol induction [26]. LB and RB in pCMFC1 are drawn with open boxes, whereas the broken LB and RB due to T-DNA integration into plant genome are shown with hatched boxes. *T*_*Nos*_, terminator of nopaline synthesis (*Nos*) gene; *CRE-int*, bacteriophage P1 Cre recombinase gene with an intron; *O*_*LexA-46*_ *-P*_*35Smini*_, eight copies of LexA DNA binding site fused to the −46 CaMV 35S mini-promoter; *Hpt*, coding region of hygromycin phosphotransferase gene; *P*_*Nos*_, *Nos* gene promoter; *T*_*E9*_, *rbcS* E9 terminator; XVE, open reading frame encoding chimeric transactivator containing the regulator domain of an estrogen receptor; *P*_*35S*_, CaMV 35S promoter; F1, R1, F2 and R2, DNA primers used for PCR analysis to detect Cre/*loxP*-mediated DNA recombination (Table 1). Only one *Pml*I or *Pme*I cleavage site is identified in the T-DNA region. Two *Hind*III or *Xba*I cleavage sites are indicated in the map, respectively. Other *Hind*III or *Xba*I sites in the regions flanked by the two *Hind*III or *Xba*I sites are not shown. DNA probes for the *Nos* terminator (*T*_*Nos*_) or the coding region of the *Hpt* gene (*Hpt*) are indicated

The binary construct pCMFC1 (Fig. 1) was used to generate transgenic jatropha plants through *Agrobacterium*-mediated jatropha transformation [25]. Theoretically, the *β*-estradiol-regulated Cre/*loxP*-mediated DNA recombination system in the T-DNA region of pCMFC1 enables the removal of the hygromycin phosphotransferase gene (*Hpt*) in the *loxP* fragment after *β*-estradiol induction and the production of marker-free transgenic plants [26]. The DNA recombination in the marker-free transgenic plants could be detected by PCR analysis using a set of DNA primer pairs flanking the *loxP* sites before or after *loxP* fragment excision (Fig. 1; Table 1). In this study, marker-free T-DNA could be identified by the amplification of the F1-R2 fragment (737 bp) flanking the remaining *loxP* site after *loxP* fragment excision, while non-marker-free T-DNA, T-DNA undergone incomplete *loxP* fragment excision and truncated T-DNA can be detected by the amplification of the F1-R1 fragment (533 bp) flanking the *loxP* site next to the left border and/or the F2-R2 fragment (811 bp) flanking the *loxP* site adjacent to the *C1* promoter (Fig. 1).

**Table 1:** DNA primers used in this study

Primer name	Nucleotide sequence (5’ to 3’)
C1-ApaI-F	ATTAGGGCCCAGGTAAGCTTCAGG
C1-R	ATTGATTTCACCTGTCCAGTTGTAT
C1SP-F	GGCATCGGCTAGGGAAATAG
C1SP-R	TGCTACTTGGGTGACATTGTTC
F1	GAATTGTCGAGGTCGAAGATC
R1	ATAGTGAAACAGGGGCAATGG
F2	ACGGCGAGTTCTGTTAGGTC
R2	TCATCGGGTTTCGGTGACTC
Hpt-F1	AAAAAGCCTGAACTCACCGCGACGT
Hpt-R1	TACTTCTACACAGCCATCGGTCCA
Hpt-R4	ATGGCCTCCGCGACCGGC
T_Nos_-F	TACAAAGTGGTGATAAGGGCG
T_Nos_-R	AAACTGAAGGCGGGAAACGAC
Gus-L-F	CGCATTACCCTTACGCTGAAGAG
Gus-L-R	AGACGCGGTGATACATATCCAGC

**Fig. 2:**
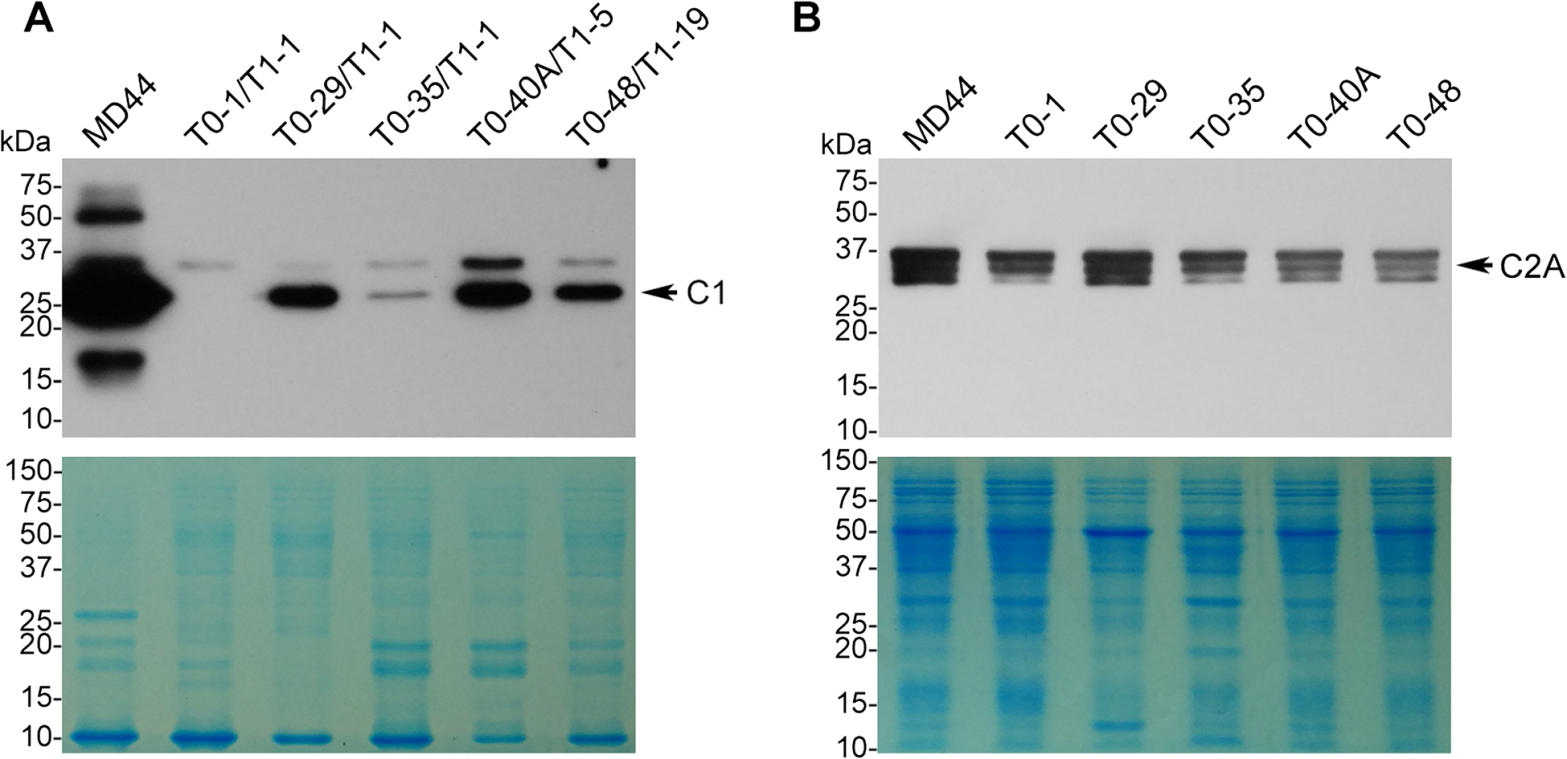
Western blot analysis of curcin proteins in transgenic jatropha plants. **a** Detection of C1 proteins in the endosperm of transgenic T1 seeds by western blot analysis. T0-1/T1-1, T0-29/T1-1, T0-35/T1-1, T0-40A/T1-5 and T0-48/T1-19 are transgenic T1 seeds carrying RNAi cassettes derived from the respective T0 plants. **b** Detection of C2A proteins in young leaves of T0 plants by western blot analysis. Proteins isolated from the endosperm of mature jatropha seeds (**a**) or young leaves (**b**) were separated by 8 % SDS-PAGE. Curcin proteins were detected by anti-C1 antibodies. Proteins stained with Coomassie brilliant blue in duplicate SDS-PAGE gels served as protein loading controls. Arrows indicate the positions of C1 (**a**) and C2A (**b**), respectively. kDa, kilodalton; MD44, non-transgenic control

**Fig. 3:**
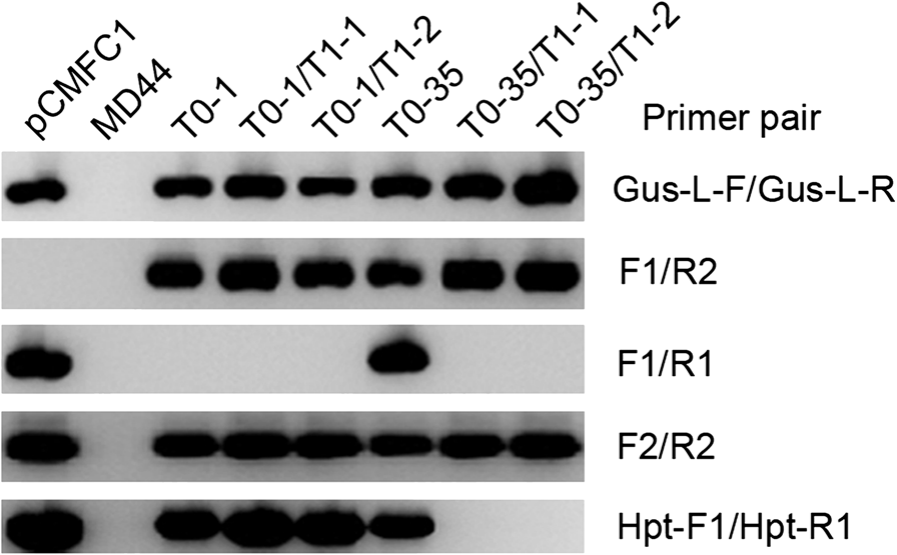
PCR analysis of T0-1 and T0-35 and their T1 progeny. The DNA sequences of primers are listed in Table 1. pCMFC1, control plasmid; MD44, wild-type control. T0-1/T1-1 and T0-1/T1-2, T1 plants derived from T0 plant T0-1; T0-35/T1-1 and T0-35/T1-2, T1 plants derived from T0 plant T0-35

In total, twelve transgenic T0 plants were obtained after *Agrobacterium*-mediated transformation of jatropha cotyledon discs [25]. Initial PCR analysis indicated that all of the twelve T0 plants carried *C1* promoter-driven RNAi cassette for the *C1* gene, showing the amplification of *Gus* linker (Table 2). However, six of the twelve T0 plants gave amplification of the F1-R2 fragment, indicating that they carried marker-free T-DNA (Table 2). The transgenic T0 plants grew and developed normally compared to wild-type MD44 in the same growth condition. T1 seeds from the T0 plants were collected and used for further molecular analysis. Embryos of the T1 seeds were dissected and germinated on seed germination medium, while the endosperm from the same set of T1 seeds was analyzed individually for C1 proteins by western blot analysis. T1 plants were transplanted to soil and used for molecular characterization of the transgenes. Initial screening identified five T0 plants, T0-1, T0-29, T0-35, T0-40A and T0-48. They produced T1 seeds that had lower C1 content than non-transgenic MD44 seeds (Fig. 2a). Among the five transgenic lines, T1 seeds derived from T0-1 and T0-35 had the lowest level of C1 content (Fig. 2a, lanes 2 and 4). Both T0-1 and T0-35 carried marker-free T-DNA, showing the amplification of F1-R2 fragment (Table 2; Fig. 3). However, PCR analysis indicated that they also carried the *Hpt* gene (Table 2; Fig. 3). The results suggested that the two T0 plants carried both marker-free and non-marker-free T-DNAs. T1 plants T0-1/T1-1, T0-1/T1-2, T0-35/T1-1 and T0-35/T1-2 inherited the marker-free T-DNAs from the respective T0 plants, showing the amplification of the *Gus* linker and F1-R2 fragments (Fig. 3). However, they also showed the amplification of F2-R2 fragment (Fig. 3). In addition, T0-1/T1-1 and T0-1/T1-2 still contained the *Hpt* gene (Fig. 3). The results suggested that the T1 plants carried either non-marker-free T-DNA or truncated T-DNA.

**Table 2:** Summary of PCR analysis for T0 transgenic plants^a^

Name	*Gus* linker	F1-R2	*Hpt*	F1-R1	F2-R2
MD44	−	−	−	−	−
T0-1	+	+	+	−	+
T0-20A	+	+	+	+	+
T0-25A	+	−	+	−	−
T0-29	+	−	+	−	−
T0-30	+	−	−	−	−
T0-33	+	+	+	+	+
T0-34	+	−	−	−	−
T0-35	+	+	+	+	+
T0-36	+	−	+	−	+
T0-40A	+	+	+	−	−
T0-40B	+	−	+	−	−
T0-48	+	+	+	+	+

^a^DNA primer pairs for PCR amplification are as follows: *Gus* linker, Gus-L-F and Gus-L-R; F1-R2, F1 and R2; *Hpt*, Hpt-F1 and Hpt-R1; F1-R1, F1 and R1; F2-R2, F2 and R2. The DNA sequences of the primers are listed in Table 1

The *C1* gene was previously identified to be only expressed in jatropha seeds. In this study, the RNAi cassette for the *C1* gene was driven by a native *C1* promoter. Previously, the *C2A* gene was found to be mainly expressed in young leaves of *J. curcas*. To investigate if its expression was affected in the *C1* RNAi plants, proteins from young leaves of the 5 transgenic T0 plants were isolated and subjected to western blot analysis. C2A with molecular size at about 30 kDa was detected and its expression level did not show significant difference in young leaves between MD44 and the transgenic T0 plants (Fig. 2b). The result indicates that the RNAi-mediated gene silencing driven by the endosperm-specific *C1* promoter did not suppress the expression of the *C2A* gene in the young leaves of transgenic jatropha plants.

### Molecular and genetic analyses of transgenic plants

**Fig. 4:**
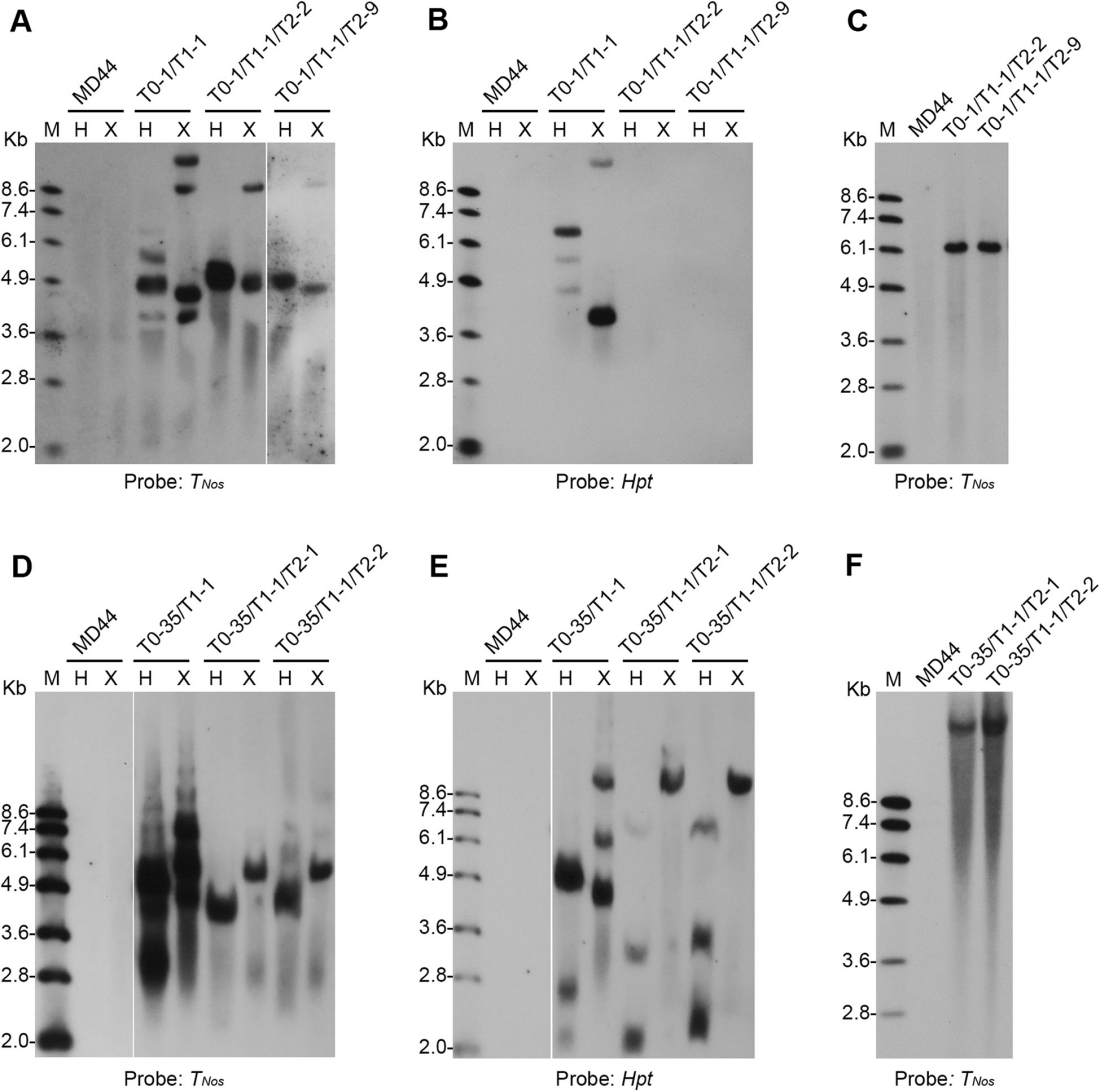
Southern blot analysis of transgenic plants. **a** to **c** Southern blot analysis of MD44 and T2 plants derived from transgenic T1 plant T0-1/T1-1. **d** to (**f**) Southern blot analysis of MD44 and T2 plants derived from transgenic T1 plant T0-35/T1-1. Plant genomic DNA was digested by single restriction enzymes *Hind*III (H) or *Xba*I (X) (**a**, **b**, **d** and **e**), or with the combination of *Pml*I and *Pme*I (**c** and **f**) and then fractionated on a 0.8 % agarose gel. Southern blots were probed with *T*_*Nos*_ (**a**, **c**, **d** and **f**) or *Hpt* (**b** and **e**) probes. M, DNA molecular marker; Kb, kilobase

Southern blot analysis using the *T*_*Nos*_ probe identified at least four hybridization bands in T0-1/T1-1 when the genomic DNA was digested by *Hind*III or *Xba*I (Fig. 4a, lanes 4 and 5). Meanwhile, two to three copies of the *Hpt* gene were detected by the *Hpt* probe (Fig. 4b, lanes 4 and 5). Considering that T0-1/T1-1 gave PCR amplification of F1-R2, F2-R2 and *Hpt* fragments (Fig. 3), the results collectively suggested that T0-1/T1-1 carried at least one copy of marker-free T-DNA and two to three copies of intact or truncated non-marker-free T-DNA. In the same Southern blot experiment, two hybridization bands were detected in T2 plants T0-1/T1-1/T2-2 and T0-1/T1-1/T2-9 by the *T*_*Nos*_ probe, respectively (Fig. 4a, lanes 7 and 9). No signal of the *Hpt* gene was detected when the same Southern blot was stripped and re-hybridized with the *Hpt* probe (Fig. 4a and b, lanes 6 to 9). The results indicated that both T0-1/T1-1/T2-2 and T0-1/T1-1/T2-9 were marker-free plants that carried marker-free T-DNA(s) only. As there is no *Xba*I digestion site in the region between the *T*_*Nos*_ probe and the right border (RB) of T-DNA and another *Xba*I site is on the jatropha genomic DNA which flanked the T-DNA, only one band would be detected by the *T*_*Nos*_ probe from each marker-free T-DNA (Fig. 1). Therefore, each marker-free T2 plant should carry two copies of marker-free T-DNA. In addition, both *Pml*I and *Pme*I have only one digestion site in the T-DNA region of pCMFC1, respectively (Fig. 1). Double digestion of T-DNA or marker-free T-DNA with *Pml*I and *Pme*I releases a 6565-bp *Pml*I-*Pme*I fragment, which includes the intact RNAi cassette (Fig. 1). Indeed, an expected 6.5-kb *Pml*I-*Pme*I band was detected by the *T*_*Nos*_ probe in the two marker-free transgenic plants, respectively (Fig. 4c). The results also confirm that the two copies of the marker-free T-DNA in T0-1/T1-1/T2-2 or T0-1/T1-1/T2-9 are intact after the *loxP* fragment excision. T0-1/T1-1/T2-2 and T0-1/T1-1/T2-9 had a similar transgene genotype and belonged to the same transgenic line. The transgenic line was designated as L1.

At least four hybridization bands were detected in T0-35/T1-1 by the *T*_*Nos*_ probe (Fig. 4d). Initial PCR analysis using primers Hpt-F1 and Hpt-R1 failed to amplify a 969-bp fragment in the 1026-bp coding region of the *Hpt* gene from T0-35/T1-1 (Fig. 3). However, at least 3 hybridization bands were detected in the T1 plants by the *Hpt* probe (Fig. 4e). The results implied that T0-35/T1-1 may carry multiple copies of truncated *Hpt* genes. The presence of truncated *Hpt* genes in T0-35/T1-1 was further verified by PCR amplification of a 353-bp fragment in the 5’ coding region of the *Hpt* gene using DNA primers Hpt-F1 and Hpt-R4 (Table 1) (data not shown). In the T2 generation, both T0-35/T1-1/T2-1 and T0-35/T1-1/T2-2 produced one major hybridization band when detected by the *T*_*Nos*_ probe (Fig. 4d, lanes 6 to 9). Southern blot analysis using the *Hpt* probe identified three hybridization bands when the genomic DNA was digested with *Hind*III, but only one band when digested with *Xba*I (Fig. 4e, lanes 6 to 9). The results indicated that the three copies of the truncated *Hpt* gene might be inserted into the same locus of jatropha genome. Further Southern blot analysis using the *T*_*Nos*_ probe identified a single hybridization band in T0-35/T1-1/T2-1 and T0-35/T1-1/T2-2, respectively, when the genomic DNA was double digested by *Pml*I and *Pme*I (Fig. 4f). However, the hybridization band had molecular size at about 20 kb, much greater than the expected 6565-bp *Pml*I-*Pme*I fragment (Fig. 4f). The result suggested that either one or both of the *Pml*I and *Pme*I sites were mutated or lost in the marker-free T-DNAs in the two T2 plants, due to illegitimate T-DNA integration or Cre/*loxP*-mediated *loxP* fragment excision. The truncated *Hpt* genes might function due to deletion of large fragment at the 3’ coding region of the *Hpt* gene. T0-35/T1-1/T2-1 and T0-35/T1-1/T2-2 belonged to the same transgenic line. The transgenic line was designated as L35.

### Silencing of *C1* gene expression in endosperm of L1 and L35

**Fig. 5:**
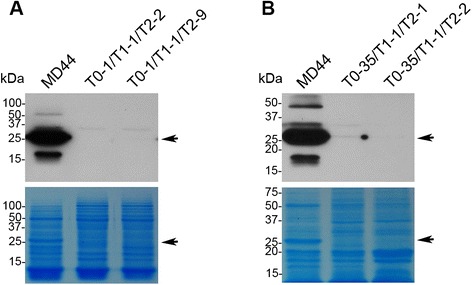
Western blot analysis of curcin proteins in the endosperm of transgenic T2 seeds of L1 and L35. **a** Western blot analysis with total proteins isolated from the endosperm of T2 seeds of L1. T0-1/T1-1/T2-2 and T0-1/T1-1/T2-9 are T2 individuals belonged to transgenic line L1. **b** Western blot analysis with total proteins isolated from the endosperm of T2 seeds of L35. T0-35/T1-1/T2-1 and T0-35/T1-1/T2-2 are T2 individuals belonged to transgenic line L35. Proteins isolated from the endosperm of mature jatropha seeds were separated by SDS-PAGE and curcin proteins were detected by anti-C1 antibodies. Proteins stained with Coomassie brilliant blue in duplicate SDS-PAGE gels serve as protein loading controls. The arrows indicate the positions of C1 in western blot analysis and SDS-PAGE gels, respectively

In the parallel experiments, C1 proteins in endosperm of T2 seeds of L1 and L35 and of non-transgenic MD44 were detected by western blot analysis using anti-C1 antibodies. A high level of C1 protein was detected in MD44 endosperm (Fig. 5a and b). The putative C1 band in the lane of MD44 endosperm was so strong that it was visible after the proteins in SDS-PAGE gel were stained with Coomassie brilliant blue (Fig. 5a and b). However, the C1 protein in L1 and L35 endosperm was weakly detected in western blot analysis (Fig. 5a and b). The results demonstrated that the RNAi cassettes in L1 and L35 were functional in silencing of the *C1* gene.

**Fig. 6:**
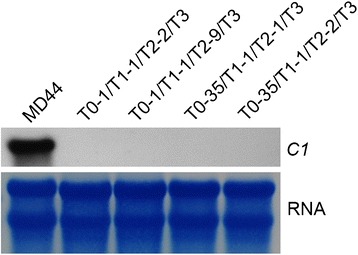
Northern blot analysis of *C1* gene transcripts in the endosperm of T3 seeds of L1 and L35. Total RNA was isolated from 6-week-old immature seeds of MD44 and T3 progeny derived from the T2 individuals of L1 (T0-1/T1-1/T2-2 and T0-1/T1-1/T2-9) and L35 (T0-35/T1-1/T2-1 and T0-35/T1-1/T2-2). rRNAs on methylene blue-stained membranes are shown as a loading control

We previously demonstrated that the *C1* transcripts were highly expressed in 6-week-old developing seeds. The 6-week-old immature T3 seeds from L1 and L35 plants were screened for the presence of RNAi cassette. Total RNA isolated from individual T3 seeds was subjected to northern blot analysis for detection of *C1* transcripts. Compared to high level of *C1* transcripts in the endosperm of non-transgenic MD44, the *C1* transcripts could not be detected in the endosperm of L1 and L35 seeds that carried the RNAi cassette (Fig. 6). The results demonstrated that the down regulation of curcin proteins in transgenic jatropha seeds resulted from RNAi-mediated *C1* gene silencing.

## Discussion

Using endosperm-specific RNAi-mediated gene silencing and *β*-estradiol-regulated Cre/*loxP* system, we have generated two independent transgenic jatropha lines that produce curcin-deficient transgenic seeds. Line L1 consisted of two T2 plants, T0-1/T1-1/T2-2 and T0-1/T1-1/T2-9, which were derived from T0 plant T0-1. L1 plants carry two copies of marker-free RNAi cassette for the *C1* gene. The two RNAi cassettes may be separated in the subsequent generations if they are not closely linked to each other. Line L35 had two T2 plants, T0-35/T1-1/T2-1 and T0-35/T1-1/T2-2, which were derived from T0 plant T0-35. L35 plants carry a single copy of marker-free RNAi cassette for the *C1* gene and three copies of closely linked but truncated *Hpt* genes. L35 plants may eliminate the truncated *Hpt* genes in subsequent generations if they could be separated from the marker-free RNAi cassette. In both transgenic lines, the functional marker-free RNAi cassettes could be used for further jatropha breeding through marker-assisted selection.

We previously demonstrated that C1 is specifically expressed and stored in the endosperm of jatropha seeds. Jatropha also produces Type II curcins that are mainly expressed in leaves [10, 12]. To prevent the function of other curcin proteins being disrupted in other plant tissues, we chose native *C1* promoter to drive *C1* inverted repeats interspersed by a *Gus* linker. Our studies on *C1* transcripts in developing endosperm and curcin proteins in mature endosperm demonstrated that the expression of the *C1* gene was efficiently suppressed or completely silenced by the *C1* promoter-driven RNAi-mediated gene silencing. Patade et al., (2014) made a 35S promoter-driven RNAi cassette for curcin genes and used *Agrobacterium*-mediated *in planta* transformation to produce transformed jatropha plants [27]. The authors reported that the transcripts of curcin precursor gene were reduced by more than 98 % to undetectable level [27]. However, the research paper did not provide any data on molecular analysis of stable insertion of T-DNA in jatropha genome, biochemical analysis on curcin proteins in the leaves and seeds of transformed plants. Furthermore, no genetic analysis or data was given on transmission of the 35S promoter-driven RNAi cassette from the putative transformed plants to their progeny. The C1 proteins in endosperm of transgenic seeds produced in this study were weakly detected by western blot analysis. In contrast, the content of C2A, a curcin protein specifically expressed in young leaves of *J. curcas* was not affected in the T0 plants of the two lines by the endosperm-specific RNAi-mediated gene silencing for the *C1* gene. Considering the possible involvement of C2A in plant growth and development and its function in response to biotic or abiotic stress [12], its unchanged content in leaves would imply a smaller impact on the transgenic plants. Previously, two additional unknown proteins with one at about 35 kDa and another at about 17 kDa were identified in endosperm of *J. curcas* by anti-C1 antibodies in Western blot analysis. Interestingly, the content of these two proteins was reduced or silenced in the two RNAi lines, indicating that they may be curcin-related proteins or derivatives and were silenced by the RNAi cassette for the *C1* gene.

The chemically regulated, Cre/*loxP*-mediated DNA recombination system is an efficient inducible DNA recombination that has been used to generate marker-free transgenic plants in Arabidopsis [26], rice [22, 23] and *J. curcas* [24]. Although the efficiency of Cre/*loxP*-mediated DNA recombination is high, the rate of obtaining marker-free transgenic plants can be dramatically reduced by incomplete *loxP* fragment excision, and by multiple and/or truncated T-DNA insertion [22, 24]. In this study, 10 T0 plants were identified to carry marker-free T-DNA(s) after *loxP* fragment excision. However, all of them carry additional non-marker-free or truncated T-DNA. As a result, the marker-free plants were only identified in the subsequent generations. For this study, the *β*-estradiol induction for Cre/*loxP*-mediated DNA recombination was performed with regenerated hygromycin-resistant shoots rather than with hygromycin-resistant calli before regeneration. In this scenario, *β*-estradiol might not efficiently access to all types of cells, especially meristem and germline cells in the regenerated shoots. For future study, the *β*-estradiol induction can be performed with hygromycin-resistant calli before regeneration.

## Conclusion

Using endosperm-specific RNAi-mediated gene silencing and *β*-estradiol-regulated Cre/*loxP* system, we have developed marker-free transgenic jatropha plants that produce curcin-deficient seeds. The *C1* promoter-driven RNAi cassette for the *C1* gene in transgenic plants was functional and heritable. Both *C1* transcripts and C1 proteins were greatly down-regulated or silenced in the endosperm of transgenic plants. The marker-free transgenic plants and curcin-deficient seeds developed in this study provided a solution for the toxicity of curcins in jatropha seeds and addressed the safety concerns of marker genes in transgenic plants on the environment.

## Methods

### Plant materials and growth condition

*J. curcas* MD44, an elite accession widely grown in Indonesia, was used for plant transformation. MD44 and transgenic plants were grown in greenhouse at temperatures of 30 to 33 °C during the day and 24 to 26 °C at night, 85 % relative humidity and photoperiod of 12 to 13 h. The pollinated flowers and fruits were wrapped in waxed paper bags and grown till mature.

### Construction of pCMFC1

The binary RNAi construct pCMFC1 for the *C1* gene was made based on the pANDA vector [28] and pCCreloxPBt, which harbours a chemically regulated Cre/*loxP* system for the excision of marker gene [22]. Briefly, a 3765-bp promoter of the *C1* gene was amplified from BAC clone 121E10 (Accession no.: GQ925454) using *Pfu* polymerase with primers C1-ApaI-F and C1-R (Table 1) and the PCR products were digested with *Apa*I. The *Apa*I and *Sac*I fragment of the RNAi Gateway cassette in pANDA was isolated and blunted with T4 polymerase. The pCCreloxPBt plasmids were cut with *Xho*I, blunted with T4 polymerase and then digested with *Apa*I. The purified vector fragments were fused with the *Apa*I-digested *C1* gene promoter fragments and the blunt-end *Apa*I-*Sac*I fragments of the empty RNAi cassette to generate destination vector pCC1MF-GW. A partial cDNA of the *C1* gene containing a 808-bp 3’ coding region and a 54-bp 3’UTR was amplified from a *C1* cDNA clone by PCR, and cloned into pENTR D-TOPO (Invitrogen, Carlsbad, CA92008, USA), and then transferred into pCC1MF-GW to generate pCMFC1 using Gateway Technology [29]. pCMFC1 was verified by DNA sequencing. The detailed structure of the genes in the T-DNA region of pCMFC1 is showed in Fig. 1. pCMFC1 was introduced into *Agrobacterium tumefaciens* strain AGL1 by electroporation [30].

### *Agrobacterium*-mediated transformation of *J. curcas*

*Agrobacterium*-mediated transformation of *J. curcas* MD44 was performed as described previously [25]. Briefly, the cotyledon discs at the size of 0.3 × 0.3 cm^2^ were co-cultivated with *A. tumefaciens* strains AGL1 harbouring pCMFC1 on co-cultivation medium for 2–3 days at 24 °C in darkness. The co-cultivated cotyledon discs were rinsed thoroughly with sterile water and then with suspension medium containing 300 mg/L cefotaxime. Cotyledon discs were cultured on callus formation medium containing 3.5 mg/L hygromycin at 26–28 °C in darkness for 3 weeks. The cotyledon discs carrying newly emerged hygromycin-resistant calli were transferred onto shoot regeneration medium I containing 3.5 mg/L hygromycin and cultured for 3 weeks at 26–28 °C under 16-h light/8-h dark cycles. The regenerated shoots were sub-cultured on shoot regeneration medium II containing 4 mg/L hygromycin. The hygromycin-resistant shoots at about 2–3 mm were transferred onto *β*-estradiol induction medium without hygromycin to induce marker excision. After 2 weeks, the *β*-estradiol-treated shoots were transferred back to the shoot regeneration medium II without hygromycin. After 4 weeks, the regenerated shoots were transferred onto shoot elongation medium for elongation and bud multiplication. The elongated shoots at about 3-cm length were rooted on rooting medium. The putative transgenic plants with healthy root system were eventually transplanted into soil in pots at the greenhouse.

### Detection of Cre/*loxP*-mediated *loxP* fragment excision by PCR analysis

PCR analysis for the verification of transgenes and Cre/*loxP*-mediated DNA recombination in transgenic plants was conducted following the methods described previously [22].

DNA primers (F1, R1, F2 and R2) used for PCR analysis to detect Cre/*loxP*-mediated *loxP* fragment excision are listed in Table 1.

### Southern blot analysis

Jatropha genomic DNA was isolated from leaves or endosperm tissues according to the methods described previously [31]. About 2–5 μg of DNA was digested with restriction enzymes, separated on 0.8 % agarose gel and then blotted to Hybond^TM^-N^+^ nylon membrane (Amersham Biosciences, Little Chalfont, Buchinghamshire, UK). Southern blots were hybridized with DIG-labelled DNA probes for the terminator of nopaline synthesis (*Nos*) gene (T_Nos_) and the hygromycin phosphotransferase gene (*Hpt*), respectively, according to standard protocols. The primer pairs for amplification of DNA probes were T_Nos_-F/T_Nos_-R for *T*_*Nos*_ probe and Hpt-F1/Hpt-R1 for *Hpt* probe, respectively (Table 1).

### Northern blot analysis

Total RNA was isolated from jatropha endosperm using methods described previously [32]. About 10 μg total RNA was fractionated on a 1.2 % formaldehyde agarose gel and blotted onto a Hybond^TM^ N^+^ membrane (Amersham Biosciences, Little Chalfont, Buchinghamshire, UK). The DNA probe for the *C1* gene (C1 probe) for northern blot analysis was the PCR products amplified from jatropha genome with primers C1SP-F and C1SP-R (Table 1). The northern blot hybridization and the labelling of the C1 probe were similar to the methods described for the Southern blot analysis.

### Western blot analysis

Total proteins were isolated from jatropha endosperm with a homogenization buffer [0.1 M Tris–HCl, pH8.0, 0.01 M MgCl2, 18 % (*w/v*) sucrose, 40 mM *β*-mercaptoethanol]. Protein concentration was determined with Bradford’s method [33]. About 10 μg of each protein sample was separated by sodium dodecyl sulfate polyacrylamide gel electrophoresis (SDS-PAGE, 8 %), followed by blotting onto PVDF membranes (Bio-Rad, Hercules, California, USA). The C1 proteins in jatropha endosperm were detected with in-house anti-C1 polyclonal antibodies and horseradish peroxidase-coupled secondary antibodies (Bio-Rad, Hercules, California, USA) according to the product manual. Protein ladders (#SM0671, Fermentas, Glen Burnie, MD, USA) were loaded to mark molecular size of the proteins. Proteins stained with Coomassie brilliant blue in duplicate SDS-PAGE gels served as protein loading controls.
